# Assessment of protein synthesis rate enables metabolic profiling of resident-immune cells of the islets of Langerhans

**DOI:** 10.3389/fimmu.2025.1662986

**Published:** 2025-09-23

**Authors:** Lauar de Brito Monteiro, Anne-Sophie Archambault, Galina Soukhatcheva, Derek Dai, Jane Velghe, Yi-Chun Chen, C. Bruce Verchere, Ramon I. Klein Geltink

**Affiliations:** ^1^ Department of Surgery, University of British Columbia, Vancouver, BC, Canada; ^2^ BC Children’s Hospital Research Institute, Vancouver, BC, Canada; ^3^ Department of Pathology and Laboratory Medicine, University of British Columbia, Vancouver, BC, Canada; ^4^ Centre for Molecular Medicine and Therapeutics, University of British Columbia, Vancouver, BC, Canada; ^5^ University of British Columbia, Edwin S.H. Leong Centre for Healthy Aging, Vancouver, BC, Canada; ^6^ BC Cancer Research Centre, Department of Basic and Translational Research, Vancouver, BC, Canada

**Keywords:** SCENITH, single-cell energetic metabolism by profiling translation inhibition, resident macrophages, resident T cells, pancreatic islet, beta cell death, immunometabolism

## Abstract

Pancreatic islet-resident immune cells, such as lymphocytes and macrophages, support islet homeostasis, beta cell development, and tissue repair. In pathological states, including diabetes, islet immune cells can trigger inflammation, causing beta cell dysfunction and death. There has been growing interest in understanding the dynamics between beta cells and resident immune cells. Studying metabolic adaptations in beta cells and immune cells is challenging due to the mixed cell populations in islets and limited cell number, which are not suitable for conventional approaches, such as metabolomics and extracellular flux analysis. We implemented a puromycin-based flow cytometry assay for parallel analysis of the phenotype and metabolic state of islet-resident immune cells. Islets were isolated from healthy mice and exposed to a cytokine cocktail (IL-1β, TNF-α, IFN-γ) to mimic a pro-inflammatory diabetogenic microenvironment. We found that Islet-resident macrophages show higher expression of CD86 and lower expression of CD301 upon cytokine treatment, which was accompanied by reduced protein synthesis rates upon inhibition of glycolysis and mitochondrial complex V. In insulin-producing beta cells, inhibition of mitochondrial complex V (ATP synthase) by oligomycin reduces translation rates. Streptozotocin (STZ)-induced beta cell death promoted accumulation of macrophages in the islet and higher frequency of CD86^+^ macrophages, as was observed *in vitro*. Islet macrophages from STZ-treated mice showed higher basal protein synthesis rates and enhanced sensitivity to oligomycin. We validated this method in bone marrow-derived macrophages and the MIN6 beta cell line, using extracellular flux analysis as a control for the puromycin-based assay. We propose our implementation of a puromycin-based assay as a useful tool to study metabolic demands in rare islet cell populations. Applying phenotypic and protein synthesis assays coupled with specific metabolic pathway inhibitors to intact pancreatic islets can provide a better understanding of the immunometabolic cues that lead to beta cell dysfunction and failure in diabetes.

## Introduction

Pancreatic islet immune cells play an important role in beta cell development, islet homeostasis, and disease progression. Islet immune cells are a rare population, representing approximately 2-3% of total islet cells, comprising mainly macrophages, dendritic cells, and CD4 and CD8 T lymphocytes (1). Immune cell function is tightly linked to metabolic programming. Additionally, immune cells can respond to environmental metabolic cues, such as nutrient abundance or scarcity (2, 3). In the islet endocrine compartment, beta cells and alpha cells rapidly respond to changes in glucose concentration and nutrient availability to precisely regulate release of insulin and glucagon, respectively (4). Other endocrine cells in the islet, including delta, epsilon, and pancreatic polypeptide cells, are present at lower frequencies but also play a role in the control of blood sugar, metabolism, appetite, and digestion (4, 5). The pancreatic islet microenvironment experiences glucose fluctuations and hormone secretion during feeding, exposing islet-resident immune cells to changes in their extracellular environment that are unique to the pancreatic milieu. Although the role of extracellular nutrient availability and intracellular metabolic remodeling is studied in many diseases and tissue, little is known about the metabolism of resident immune cells in the islet.

When macrophages are stimulated *in vitro* with lipopolysaccharide (LPS), a component of the outer membrane of Gram-negative bacteria, and the pro-inflammatory cytokine interferon gamma (IFNγ), these cells switch to glycolysis for rapid ATP production. The tricarboxylic acid (TCA) cycle is also impaired, with flux discontinuity at isocitrate dehydrogenase and succinate dehydrogenase that leads to accumulation of (iso)citrate, succinate, and itaconate, which in turn signal to sustain inflammatory signature (6–11). On the other hand, following alternative activation of macrophages *in vitro* using IL-4, glucose fuels the TCA cycle, generating ATP via oxidative phosphorylation (OXPHOS) (6, 7). Cellular metabolic fitness is also a determinant for functionality and metabolic priming of T cells can boost their immune function *in vitro* and *in vivo* (12, 13).

When studying cellular metabolism, several techniques are routinely used to assess metabolic pathway utilization and metabolite abundance. These include mass-spectrometry-based assays, which can be used to analyze metabolite pools. Stable isotope-labeled carbon tracing can be employed to further assess specific allocation of nutrient-derived carbons, such as glucose, towards glycolysis or the mitochondrial TCA cycle. Extracellular flux analysis can be performed in live cells to assess cellular metabolism and adaptations upon metabolic challenges in real time. However, in the context of islet-resident immune cells, using these techniques is extremely challenging due to difficult access to islets *in situ*, cellular heterogeneity, and most importantly limited cell number. Transcriptomic analysis of cell-sorted macrophages from mouse islets revealed increased expression of gene sets related to OXPHOS, lipid metabolism, and lysosomal activity following STZ-induced beta cell death (14). Information at the mRNA level can provide insight into cellular dysfunction that contributes to or results from diabetes pathogenesis, but it does not account for post-transcriptional regulation of metabolic enzymes and therefore cannot accurately determine metabolic pathway activities related to immune processes. Single-cell RNA sequencing (scRNA-seq) has opened doors for thorough characterization of islet-resident immune cell signatures and is a strong tool for islet studies because it can efficiently segregate immune cell pools (macrophages, T cells, B cells, mast cells) and highlight distinct gene expression signatures in health and diabetes (15, 16). However, these approaches often require extensive validation (17) and unlike spatial transcriptomics, spatial metabolomic strategies, although promising, are expensive and lack sensitivity and single cell resolution (18). There is an urgent need for improved techniques that can accurately determine metabolic differences in rare cell populations and that consider the tissue niche in which they reside.

Development of flow cytometry-based assays that use puromycin as a readout for protein synthesis and metabolic profiling has opened new doors for *ex vivo* metabolic profiling at the single-cell level (19, 20). Single-cell energetic metabolism by profiling translation inhibition (SCENITH) integrates principles of flow cytometry and pharmacological metabolic pathway inhibition for cell-specific analysis of metabolic activity (20). This makes it possible to study metabolic adaptations in heterogeneous and rare populations. ATP production can occur in the cytoplasm (glycolysis) and in the mitochondria (OXPHOS), using metabolic intermediates generated from glucose, amino acids, or fatty acids. Among several cellular processes that consume ATP, protein synthesis is the most energy consuming (21). SCENITH uses puromycin, an analogue of aminoacylated tRNA, which delivers amino acids to elongating protein chains in the ribosome (22). Puromycin becomes incorporated during translation of newly formed proteins and is used to quantify protein synthesis rates using a fluorescently labeled anti-puromycin antibody, after a short incubation period to circumvent confounders related to complete protein synthesis inhibition (20, 23). Treatment with specific metabolic pathway inhibitors can be used to determine the contribution of cytoplasmic or mitochondrial ATP generation in steady-state and stress conditions. SCENITH has recently been used in studies of rare cell populations in tumours (20, 24), sites of cell maturation (25), and whole blood samples (20). Here, we demonstrate the use of an adaptation of SCENITH, a puromycin-based assay, to characterize phenotypic and metabolic changes in islet immune cell populations in steady state and under diabetogenic conditions.

## Materials

### Primary islet culture and cytokine treatment

- Multi-well cell culture plates – non-tissue culture-treated.
- 1640 RPMI glucose [+] L-glutamine [+], supplemented with 10% heat-inactivated fetal bovine serum (FBS), 100 U/mL penicillin-streptomycin, and 1% GlutaMAX (Gibco #35050061).
- Recombinant murine interleukin (IL)-1beta (Peprotech #211-11B).
- Recombinant murine tumor necrosis factor (TNF) alpha (Peprotech #315-01A).
- Recombinant murine interferon (IFN) gamma (Peprotech #315-05).
- Isolated islets from BL6 mice, according to previous protocol (26). C57BL/6 mice were purchased from the Jackson laboratory (Bar Harbour, USA) and kept at the BC Children’s Hospital Research Institute Animal Facility (protocol # A24-0113), in compliance with applicable guidelines. Female and male mice aged 8–20 weeks were used for experiments.
- .Human islets were provided by the University of Alberta Islet Core (Human islet ethics protocol #H20-01786).

### Bone marrow-derived macrophage polarization

- Bone marrow-derived macrophages (BMDMs).
- Recombinant murine IFN gamma (Peprotech #315-05).
- Recombinant murine IL-4 (Peprotech #214-14).
- Lipopolysaccharides (LPS) from *Escherichia coli* (Sigma #L2630).

### Treatment with metabolic inhibitors to assess cellular metabolism

- 2-Deoxy-D-glucose (Sigma #D8375) – 2-Deoxy-D-glucose (2-DG) is a glucose analogue that inhibits glycolysis and is non-metabolizable.
- Oligomycin (Sigma #O4876) – Oligomycin is an inhibitor of mitochondrial Complex V of the electron transport chain, also known as ATP synthase.
- Cycloheximide solution (Sigma #C4859) – Cycloheximide inhibits protein translation elongation and is used in this assay as a control of protein synthesis inhibition.
- Puromycin (Sigma #P8833) – Puromycin inhibits protein synthesis by being incorporated into the C-terminus of nascent protein chains, halting protein translation (8, 22).

### Islet dispersion

- TrypLe Express (Gibco #12604021) – used for human and mouse islet dispersion*.
  *Accumax (Invitrogen #00-4666-56) – alternative for primary islet dispersion. Used for stem cell-derived pseudo-islet dispersion.
- PBS + EDTA solution: Mg^2+^ and Ca^2+^ free phosphate buffered saline (PBS) + 0.5 mM EDTA.
- FACS buffer: PBS + 2% FBS.
- Cell culture incubator at 37 °C and 5% CO_2_.
- FACS tubes - Falcon^®^ round-bottom polystyrene tubes, 5mL (VWR #CA60819-820).
- Ice bucket

### Cell staining and flow cytometry

- FACS Buffer: PBS + 2% FBS.
- Antibodies for cell surface markers staining – suggested panel in **Table 1**.
- eBioscience™ Foxp3/Transcription Factor Fixation/Permeabilization Concentrate and Diluent (Invitrogen #00-5521-00).
- eBioscience™ Permeabilization Buffer 10X (Invitrogen #00-8333-56).
- Antibodies for intracellular markers staining – suggested panel in **Table 1**.

**Table 1:** Panel for islet endocrine/immune cell populations by flow cytometry.

Mouse reactivity				
Target/probe	Dilution	Fluorochrome	Clone	Cat. number
Anti-mouse CD45	1:200	Brilliant Violet™ 605 Excitation/emission: 405/603	30-F11	BioLegend #103155
Anti-mouse F4/80	1:200	Super Bright™ 702 Excitation/emission: 413/702	BM8	Invitrogen #67-4801-82
Anti-mouse CD3	1:200	Brilliant Violet™ 510 Excitation/emission: 405/510	17A2	BioLegend #100234
Anti-mouse CD86	1:200	Alexa Fluor 700 Excitation/emission: 633/719	GL-1	BioLegend #105024
Anti-mouse CD206*	1:200	Brilliant Violet™ 421 Excitation/emission: 405/421	C068C2	BioLegend #141717
Human reactivity				
Target/Probe	**Dilution**	**Fluorochrome**	**Clone**	**Cat. number**
Anti-human CD45	1:200	Brilliant Violet™ 605 Excitation/emission: 405/603	HI30	BioLegend #304042
Anti-human CD68	1:200	Brilliant Violet™785 Excitation/emission: 405/785	Y1/82A	BioLegend #333826
Anti-human CD3	1:200	PerCP-Cy5.5 Excitation/emission: 482/690	OKT3	BioLegend #317336
Mouse and human reactivity				
Target/Probe	**Dilution**	**Fluorochrome**	**Clone**	**Cat. number**
eBioscience™ Fixable Viability Dye	1:10000	eFluor780 Excitation/emission: 633/780		Invitrogen #65-0865-18
Anti-mouse and human insulin	1:50	Alexa Fluor 647 Excitation/emission: 653/669	T56-706	BD Biosciences #565689
Anti-mouse and human glucagon*	1:200	Brilliant Violet™ 421 Excitation/emission: 405/421	U16-850	BD Biosciences #565891
Anti-Puromycin	1:2500	Alexa Fluor 488 Excitation/emission: 488/496	12D10	Sigma #MABE343-AF488

*These antibodies cannot be used in the same panel.

### Extracellular flux analysis

- Seahorse XFe96 Bioanalyzer (Agilent Technologies).
- Agilent Seahorse XF Pro FluxPak (Agilent #103792-100), contains:
  - 500mL bottle of XF Calibrant (part #100840-000).
  - 18 XFe96/XF Pro M Cell Culture Microplates (part #103774-100).
  - 18 XFe96/XF Pro sensor cartridges.
  - 3 pairs of XF Loading Guide.
- Bone marrow-derived macrophages (BMDMs)*.
- MIN-6 cells (AddexBio #C0018008) – Mouse insulinoma cell line*.
- AddexBio Advanced DMEM Medium (AddexBio #C0003-04) supplemented with 15% FBS and 0.05mM 2-marcaptoethanol (Invitrogen #21985023) – optimized medium for MIN-6 growth*.
*BMDMs and MIN-6 cells were used in this protocol for validation of the puromycin-based cellular metabolism assay.
- XF media: unbuffered 1640 RPMI supplemented with 1mM pyruvate, 2mM L-glutamine, pH7.4 (27).
- PBS.
- Glucose (Sigma #G7021).
- 2-Deoxy-D-glucose (Sigma #D8375).
- Oligomycin (Sigma #O4876).
- Carbonyl cyanide 4-(trifluoromethoxy)phenylhydrazone (Sigma #C2920) – also known as FCCP, this is an uncoupler of oxidative phosphorylation causing mitochondrial membrane depolarization.
- Rotenone (Sigma #R8875) – inhibitor of nicotinamide adenine dinucleotide (NADH):ubiquinone oxidoreductase (mitochondrial complex I of the electron transport chain).
- Antimycin A (Sigma #A8674) – inhibitor of coenzyme Q-cytochrome c reductase (mitochondrial complex III of the electron transport chain).
- Non-CO2 incubator (no fan) at 37°C.

### Induction of beta cell death with STZ

- Streptozotocin (STZ; Sigma #S0130) - targets beta cells specifically via the GLUT2 glucose transporter and causes DNA damage and beta-cell death.
- 1mL Insulin Syringes (BD #329420) with 0.36mm (28G) needle, for intraperitoneal (i.p.) injections.
- STZ buffer: 0.2M sodium acetate solution, pH 4.5.
- 5% KOH solution.
- OneTouch Ultra^®^ glucose test strips.
- OneTouch Ultra^®^2 meter – blood glucose monitor.
- Precision balance scale.
- 1.5 mL Eppendorf tubes.

### Software

All statistical analysis was generated using Prism 10 (GraphPad Software). Schematics were generated using BioRender. Flow cytometry plots were analyzed with FlowJo software (BD).

## Methods

### Primary islet cytokine treatment

**Figure 1:**
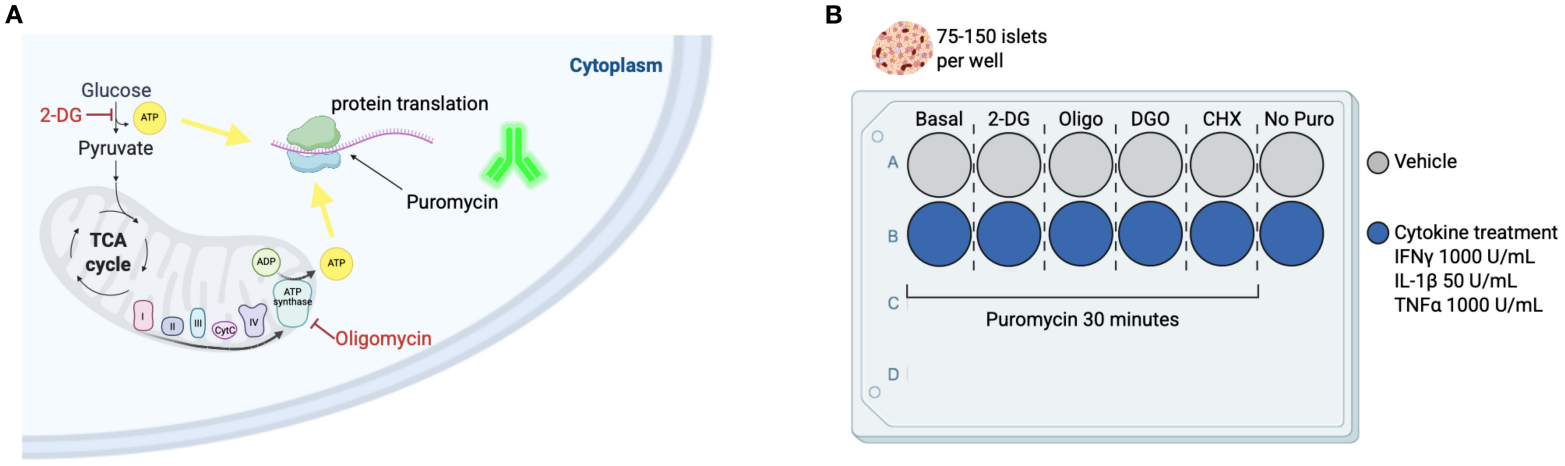
Using puromycin as a readout for metabolic pathway reliance upon pharmacological disruptions. **(A)** In this method adaptation, we implement principles of SCENITH (20) to study metabolic pathway usage for energy production in pancreatic islet endocrine and immune cell populations. This study provides an in-depth methodology optimized for mouse and human islets. We apply a puromycin-based approach and later disperse primary islets and use the cells in suspension for flow cytometry acquisition and determination of metabolic features in distinct cell types within the islet. ATP derived from glycolysis and ATP synthase are consumed during protein translation. Inhibition of glycolysis using 2-deoxyglucose (2-DG) can determine the role of glycolysis-derived ATP for protein synthesis (PS). Oligomycin (Oligo) inhibits mitochondrial complex V of the electron transport chain (ATP synthase) and prevents mitochondrial ATP production, thus providing a readout for reliance on mitochondrial respiration for PS. Puromycin is an analogue of aminocylated-tRNA and gets promptly incorporated into a nascent protein chain during translation, so a fluorescent anti-puromycin antibody is used to determine puromycin incorporation rates, or PS rates. **(B)** Suggested plating guide for n=1. We suggest splitting the collect islets into 75–150 islets per well for pre-treatments with DMSO vehicle control or pro-inflammatory cytokines, and also for pharmacological inhibitors of metabolic pathways (for determination of basal PS rates, and PS rates following 2-DG, Oligo, 2-DG+Oligo [DGO], and cycloheximide [CHX] – a PS inhibition control). Including a ‘No puromycin’ control as a negative control is recommended. This should be repeated for as many replicates as your experiment requires. It is not always viable to split islets from a single mouse into all these conditions, so either pooling biological replicates or limiting the number of conditions, e.g. Basal, 2-DG, and Oligo, can provide insights into energy usage by islet endocrine and resident immune cells.

1. Consider this a ‘pre-treatment’ to the later acute treatment with the metabolic inhibitors and puromycin (See **Figure 1** for suggested plating guide).
2. Let hand-picked islets rest overnight in 100 mm Petri dishes containing primary islet RPMI medium in an incubator at 37°C, with 5% CO_2_. After overnight resting, pick and plate 75–150 islets into each well of a non-treated cell culture plate, adjusting the volume of media according to your well size.
3. Treat the appropriate number of wells with either vehicle control (PBS) or a pro-inflammatory cytokine cocktail (IFN gamma 1000 U/mL, IL-1beta 50 U/mL, TNF alpha 1000 U/mL) for 24 hours.

### Treatment of primary islets with metabolic inhibitors

1. See **Figure 1** for suggested treatment time and plating guide.
2. One well of cytokine/vehicle pre-treated islets will be treated with vehicle control and used for basal levels of puromycin incorporation.
3. Treat primary islets with 2-DG to a final concentration of 100mM for a total of 15 minutes at 37°C.
4. Treat primary islets with oligomycin to a final concentration of 1.5μM for a total of 10 minutes at 37°C.
5. We recommend using cycloheximide - an inhibitor of protein synthesis as a negative control for background puromycin incorporation. For this, treat primary islets with Cycloheximide to a final concentration of 28μg/mL for a total of 15 minutes at 37°C.
6. Following the treatment with metabolic inhibitors, add puromycin to a final concentration of 10μg/mL for 30 minutes at 37°C. Do not add puromycin to the ‘No Puro’ control (See **Figure 1**).

### Islet dispersion

1. Transfer islets into a FACS tube and centrifuge the suspension for 1 min at 300 x g.
2. Gently remove supernatant without disturbing the pellet and resuspend islets in 500mL pf PBS + EDTA solution. Spin again for 1 min at 300 x g.
3. Gently remove supernatant and add 300μL of TrypLE (or Accumax). Resuspend islets by pipetting up and down several times. Incubate for 3 minutes at 37°C.
4. Remove tubes from incubator and pipette up and down again to check that the islets are dispersed and there are no clumps visible. If necessary, incubate again for 3 minutes at 37°C and repeat the process of pipetting up and down until islets are fully dispersed into a cell suspension.
5. Stop digestion reaction by adding 3mL of FACS buffer. NOTE: If there are too many samples, keep tubes that have already received FACS buffer on ice while working on the remaining samples.
6. Centrifuge tubes for 5 min at 400 x g and 4°C.
7. Remove supernatant and resuspend cells in 200μL of FACS buffer.

### Islet cell staining and flow cytometric acquisition

1. Prepare **cell surface** antibody cocktail + Fixable Viability Dye in FACS buffer to the appropriate volume of samples. NOTE: anti-Puromycin, anti-Insulin, and anti-Glucagon are stained intracellularly.
2. Centrifuge tubes containing islet cell suspension for 5 min at 400 x g and 4°C.
3. Resuspend cells in 100μL of surface antibody cocktail + Fixable Viability Dye. Incubate at 4°C in the dark for 20 minutes.
4. While cells are incubating, prepare the fixation/permeabilization solution, using the eBioscience™ Foxp3/Transcription Factor Fix/Perm Concentrate and Diluent (Invitrogen #00-5521-00):
  - Prepare a fresh solution by diluting 1 part of Fix/Perm concentrate with 3 parts of Fix/Perm Diluent. Calculate the volume of Fix/Perm solution needed for 200μL per sample.
5. Wash cells by adding 250μL of FACS buffer and centrifuging for 5 min at 400 x g and 4°C.
6. Resuspend cells in freshly prepared Fix/Perm solution and incubate in the dark at 4°C for 20 minutes. In the meantime, prepare the appropriate volume of eBioscience™ Permeabilization Buffer 10X (Invitrogen #00-8333-56) by diluting the 10X solution in distilled water. Prepare the intracellular staining antibody cocktail in Perm Buffer 1X.
7. Wash cells by adding 250μL of Perm Buffer 1X and centrifuge for 5 min at 400 x g and 4°C.
8. Resuspend cells in 100μL of intracellular antibody cocktail. Incubate at 4°C in the dark for 40 minutes.
9. Wash cells again repeating step 7. Remove supernatant and resuspend cells 200-250μL of FACS buffer for acquisition in a flow cytometer. These cells were acquired using a BD FACSymphony™ A1 Cell Analyzer.

### Extracellular flux analysis

1. Day before:
  - Start by seeding 1x105 cells per well of a 96-well plate (XFe96/XF Pro M Cell Culture Microplates) with 100μL per well as the final volume - this is for adherent cells, such as BMDMs and MIN6 cells. Use appropriate cell media for your cell of interest (See Methods). NOTE: Keep a minimum of 4 wells empty of cells to be used as background controls.
  - Let the cells rest in an incubator at 37°C, with 5% CO2 for a minimum of 3 hours if working with macrophages, or overnight if working with MIN6, until the cells have adhered to the plate.
  - Follow your protocol guide for treatments. Here, we followed BMDM polarization and cytokine treatment (See Methods).
  - Hydrate the XFe96/XF Pro sensor cartridge overnight at 37°C non-CO2 incubator by adding 180μL of XF Calibrant to each well and closing the lid of the cartridge.
2. Day of assay run:
  - Gently remove the cell culture media from wells containing BMDMs or MIN6 cells and wash wells once with 100μL of pre-warmed XF media.
  - Remove media and add 180μL of pre-warmed XF media as a final volume. Place cells in non-CO2 incubator for 45 minutes, no longer than 60 minutes.
  - Take cartridge from the non-CO2 incubator and load drugs* into appropriate ports:
    - 2-deoxyglucose (2-DG): 500mM stock; 100mM final concentration.
    - Oligomycin (Oligo): 1mM stock; 1μM final concentration.
    - FCCP: 1.5mM stock; 1.5μM final concentration.
    - Rotenone (Rot): 100μM stock; 100 nM final concentration.
    - Antimycin A (AA): 1 mM stock; 1 μM final concentration.

*Drugs can be diluted in XF media for injection into the ports of the cartridge.

d. Load cartridge into Seahorse XFe96 Bioanalyzer to calibrate. This takes about 25 minutes.

TIP: Perform steps c and d while the cells acclimatize in non-CO2 incubator.

e. Customize the Seahorse running protocol according to your experimental design. Suggestion: standard settings using 3x mixing (3 min) and measurements (3 min) cycles for baseline; inject port A - 3x mixing/measurements for 2-DG; inject port B - 3x mixing/measurement for oligomycin.

NOTE: The mitochondrial stress test (port A: oligo, port B: FCCP, port C: Rot/AA) was used to validate puromycin-based assay with BMDMs, as according to Agilent Technologies protocol.

### STZ injections

1. Day before:
  - Weigh the mice on day zero (before starting injections) to calculate the amount of STZ to be injected in each mouse.
  - Calculate injections of 30 mg/kg of STZ in buffer without exceeding 150μL of volume for injection.
2. Day of injection:
  - Measure the weight of STZ powder necessary according to the calculations and keep it in an Eppendorf tube on ice until it is time to resuspend for injection. STZ is highly sensitive to light and not stable for long after resuspension in buffer.
  - Bring buffer and STZ powder in ice bucket to approved animal facility. Bring mice cages to fume hood for injection.
  - Resuspend STZ powder in appropriate volume of buffer, shake well (vortex for about 1 min). Keep solution on ice and protected from light. Start a timer. NOTE: Since STZ is not stable in solution for very long, try to predict whether injections will take more than ten minutes. If needed, prepare multiple aliquots of STZ powder to guarantee every mouse receives fresh and stable STZ for your experiment.
  - Perform i.p. injections. The STZ group should receive the appropriate volume of freshly prepared STZ solution, and the control group should receive the appropriate volume of STZ buffer. NOTE: All STZ waste should be neutralized with 5% KOH and left in sealed container for 48 hours at room temperature.
  - Follow your local animal facility protocol for dealing with cytotoxic treatments and chemical hazards.
  - Repeat steps b-e for 5 consecutive days.
3. Days post injection:
  - Keep injected mice under close observation for 72 hours after the last injection. Keep track of mice body weight and health score daily and measure blood glucose levels periodically, according to your experimental plan.
  - Once mice start developing hyperglycemia (after 5–15 days after last STZ injection), isolate the islets for ex vivo experimentation.

## Results

### Evaluation of puromycin incorporation by murine and human beta cells

**Figure 2:**
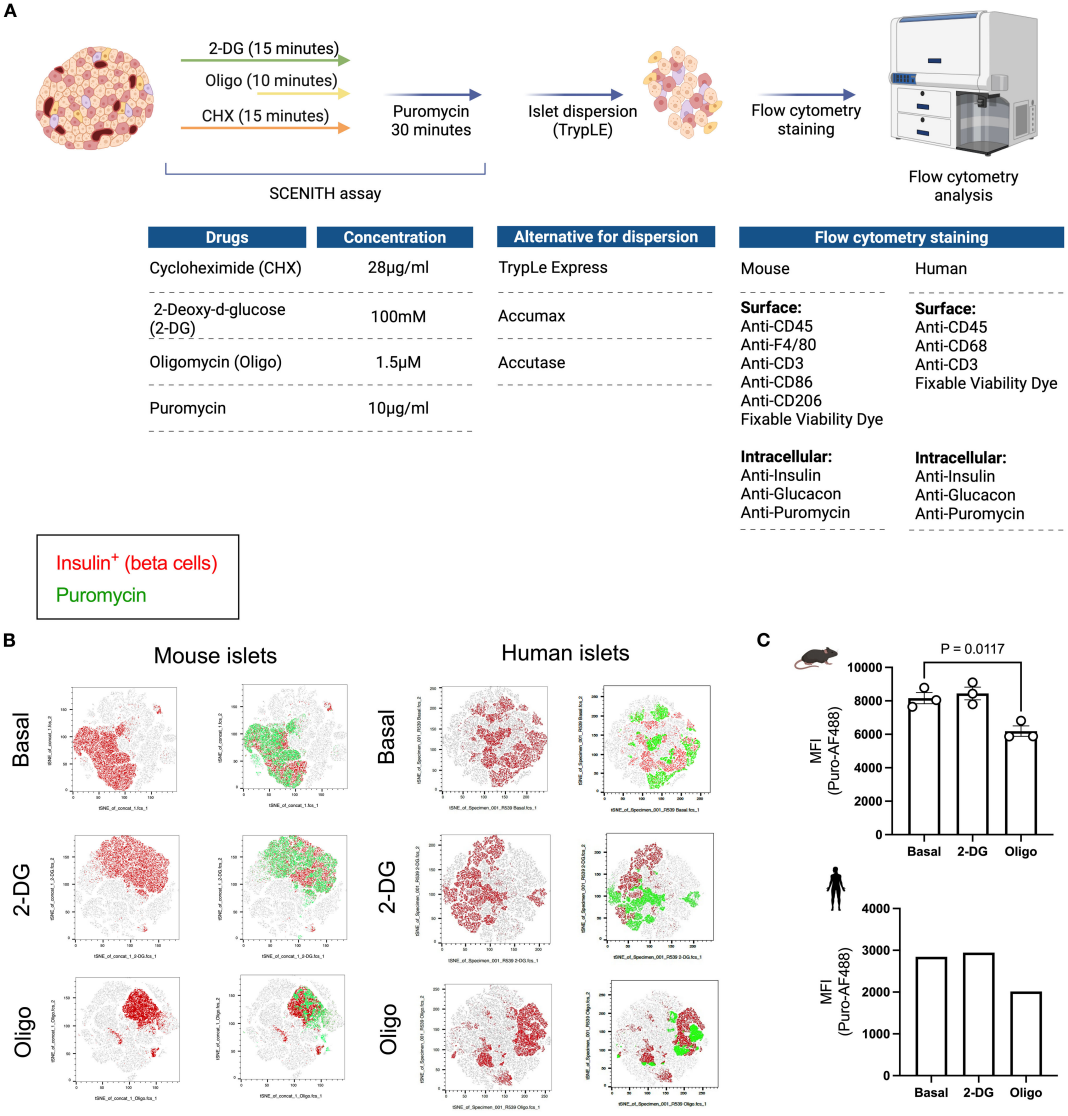
Workflow and readout of puromycin-based assay using primary islets from mice and humans. **(A)** Schematic workflow of puromycin-based assay. Whole islets (75-150) are plated into separate wells following the plating guide on **Figure 1**. 2-deoxyglucose (2-DG) and cycloheximide (Chx) are added at the same time (for a total of 15 minutes). Oligomycin (Oligo) is added 5 minutes in (for a total of 10 minutes). Then, puromycin is added in every well, except the ‘no puromycin’ control (for a total of 30 min. Islets are then dispersed and the solution obtained from the cells in suspension is stained for flow cytometry. Suggested flow cytometric panel for mouse and human samples, including surface and intracellular markers. **(B)** tSNE dimensionality reduction of beta cells (insulin^+^) in red and PS in beta cells (puromycin^+^ insulin^+^) in green shown at the basal level and following glycolysis inhibition (2-DG) and ATP synthase inhibition (oligo) from mouse islets (left panel) and human islets (right panel). **(C)** MFI of puromycin in beta cells of mouse (top) and human islets (bottom). Results are the mean +/- SEM. 3 biological replicates of mouse samples. P-values were calculated by One-Way ANOVA.

In this method adaptation (20), we used puromycin to determine protein synthesis rates in the basal state and in response to glycolysis inhibition with 2-DG or ATP synthase inhibition with oligomycin (**Figure 2A**). We used a flow cytometry-based approach to determine cell populations within the islet (**Supplementary Figure 1A**). Whole islets – murine and human (*n* = 1) – were treated with metabolic inhibitors 2-DG or oligomycin and puromycin. Islets were then dispersed into single cell suspensions for flow cytometry staining and acquisition (**Figure 2A**). The tSNE plots represent whole dispersed islets and show beta cells in red and puromycin^+^ cells in green under basal condition (vehicle-treated) and following glycolysis inhibition (2-DG) or ATP synthase inhibition (Oligo) *ex vivo* (**Figure 2B**). Mean fluorescence intensity (MFI) data (**Figure 2C**) reveal that in both mouse and human beta cells, oligomycin treatment leads to a reduction in puromycin MFI, with no effect observed after inhibition of glycolysis with 2-DG. These data suggest that, under basal non-glucose stimulated conditions, beta cells consistently rely on mitochondrial-derived ATP for protein translation (**Figures 2B, C**).

### SValidation of puromycin-based assay as a readout for beta cell metabolic profile

**Figure 3:**
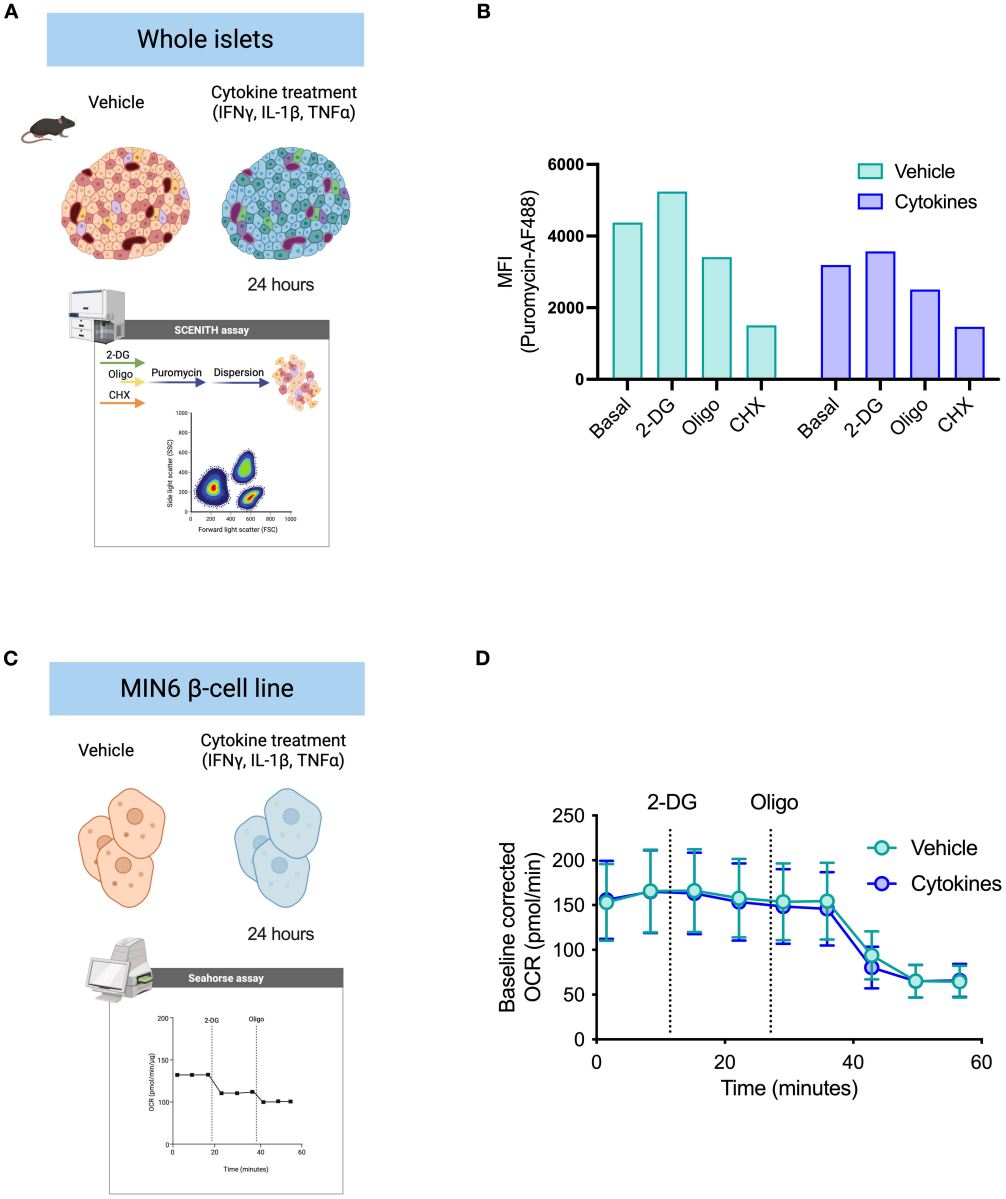
Validation of puromycin-based assay using extracellular flux analysis. **(A)** Schematic representation of whole islet treatment ex vivo prior to dispersion. Islets were treated for 24 hours with either vehicle DMSO control or a pro-inflammatory cytokine cocktail. Puromycin-based assay was performed according to the workflow suggested (see **Figure 2**). Suspension of islet cells was stained for flow cytometry acquisition. **(B)** MFI of puromycin in beta cells derived from mouse islets pre-treated with either DMSO vehicle or cytokines. Puromycin incorporation rates shown at basal level, after glycolysis inhibition (2-DG), and after ATP synthase inhibition (oligo). **(C)** Schematic representation of MIN6 cell treatment *in vitro* for extracellular flux analysis (Agilent Seahorse XF). MIN6 beta cell line (β-cell) was used for validation of beta cell metabolism. MIN6 cells were treated with either vehicle DMSO control or the same pro-inflammatory cytokine cocktail for 24 hours and submitted to a customized extracellular flux analysis assay, in which we collected OCR rates: at basal level, following 2-DG injection, and following oligomycin injection. D) Baseline-corrected OCR of MIN6 cells treated with either DMSO vehicle or cytokines. Injections of 2-DG and oligomycin (oligo) by XF analysis. Mouse islets n=3 biological replicates. MIN6 n=6 technical replicates.

Pro-inflammatory cytokines (IL-1β, TNF-α, IFN-γ) are diabetogenic stressors that accelerate diabetes progression and beta cell failure (28–30). We treated whole islets and the insulin-producing MIN6 cell line with a pro-inflammatory cytokine cocktail for 24 hours to characterize metabolic adaptations by beta cells upon cytokine stress (**Figures 3A, C**). The current gold-standard protocol for real-time cellular metabolic characterization is extracellular metabolic flux analysis. We used primary islets to perform the puromycin-based assay and MIN6 cells to determine beta cell response to metabolic inhibitors based on oxygen consumption rates (OCR) (**Figures 3A, C**), since achieving sufficient cell numbers from primary sources is a significant challenge when studying pancreatic islet cell populations. We measured puromycin MFI in MIN6 cells to characterize protein synthesis rates in response to 2-DG and oligomycin. We found a reduction in puromycin incorporation by MIN6 cells following oligomycin treatment, with a slight increase after glycolysis inhibition with 2-DG (**Supplementary Figure 2A**). Based on similar protein synthesis rates in response to inhibition of glycolysis and ATP synthase by MIN6 cells, murine beta cells, and human beta cells, we decided to perform extracellular flux analysis on MIN6 cells to compare the efficacy of the protein synthesis rates in predicting mitochondrial reliance for ATP generation in primary beta cells. We found that primary beta cells showed lower puromycin incorporation after oligomycin treatment and higher or equal puromycin MFI in response to 2-DG (**Figure 3B**). Similarly, MIN6 cells did not alter the OCR upon 2-DG injection and showed a decrease in oxygen consumption rate (OCR) after oligomycin injection (**Figure 3D**). This was independent of whether the islets or MIN6 cells had been previously treated with vehicle control or pro-inflammatory cytokine cocktail. These data suggest that beta cells maintain a robust response to oligomycin even in the presence of cytokine stress, and mitochondrial ATP-coupled respiration is not glucose dependent in these cells.

### Puromycin-based assay allows for assessment of islet-resident macrophage metabolic profiling

**Figure 4:**
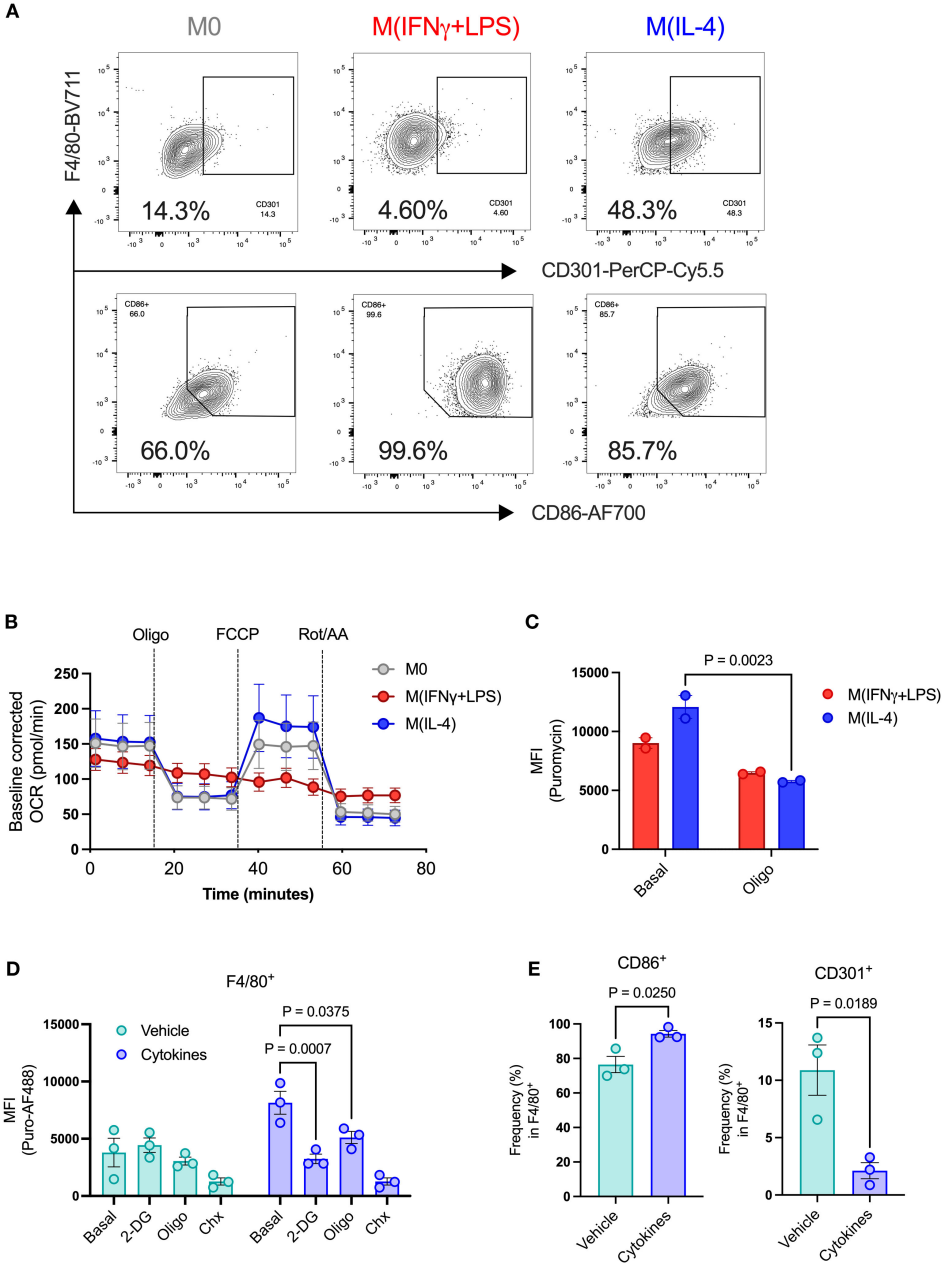
Metabolic profiling of islet resident macrophages by puromycin-based assay. BMDMs were used to validate metabolic changes in islet-resident macrophages. BMDMs were left unpolarized (M0) or submitted to 24h polarization in IFNγ+LPS or IL-4. **(A)** Expression of CD301 and CD86 in BMDMs (F4/80^+^). **(B)** Baseline-corrected OCR of BMDMs. Injections of oligomycin (Oligo), FCCP, rotenone and antimycin A (Rot/AA). **(C)** MFI of puromycin in BMDMs (F4/80^+^) at the basal level and after oligomycin treatment (Oligo). **(D)** MFI of puromycin in islet-resident macrophages (F4/80^+^) from dispersed mouse islets treated *ex vivo* with either DMSO vehicle or cytokines. PS rates (puromycin incorporation) shown at basal levels, following 2-DG, oligomycin (oligo), and cycloheximide (Chx) treatments. **(E)** Expression of CD86 and CD301 in islet-resident macrophages (F4/80^+^) from dispersed mouse islets treated *ex vivo* with either DMSO vehicle or cytokines. BMDMs n=2–6 biological replicates. Mouse islets n= 3 biological replicates. Results are the mean +/- SEM. P-values were calculated by Two-Way ANOVA **(C, D)** with Sidak’s multiple comparison test and t test **(E, F)**.

To validate our approach for assessing metabolic pathways in polarized macrophages, we treated BMDMs with IL-4 to establish a pro-resolution state, as shown by higher expression of CD301 (**Supplementary Figure 2B**, **Figure 4A**). We also used IFN-gamma plus LPS to promote a pro-inflammatory state (M[IFNγ+LPS]), evidenced by increased expression of the costimulatory molecule CD86 (**Supplementary Figure 2B**, **Figure 4A**). We then performed a mitochondria stress test by extracellular flux assay to visualize the differences in OCR in polarized macrophages. As expected, M(IFNγ+LPS) had lower basal OCR than non-polarized (M0) and M(IL-4) cells. Additionally, M(IFNγ+LPS) did not respond to oligomycin treatment, nor did they show elevated OCR after uncoupling with FCCP (**Figure 4B**). Conversely, when measuring puromycin incorporation levels, M(IFNγ+LPS) showed no significant response to oligomycin treatment, while M(IL-4) had not only higher basal incorporation levels but also presented a significant reduction upon addition of oligomycin (**Figure 4C**). M(IFNγ+LPS) also showed lower puromycin MFI compared to both unpolarized (M0) control and M(IL-4) (**Supplementary Figure 2C**). These data, along with other published studies (31) support the potential value of this protein synthesis-based assay for probing macrophage metabolic pathway activity.

We then collected pancreatic islets from healthy adult mice and measured protein synthesis following pro-inflammatory cytokine cocktail treatment. After cell dispersion, we gated the macrophage population (F4/80^+^) to evaluate protein synthesis rates in islet-resident macrophages (**Supplementary Figure 1A**). Previous studies characterized islet macrophages by CD45^+^ MHCII^+^ F4/80^+^ and showed that macrophages are a double positive population of MHCII^+^ F4/80^+^ cells (32), with no single positive population detected, thus we used F4/80 gated inside the CD45^+^ population for identification of islet macrophages. Although we were unable to detect differences in protein synthesis rates between vehicle- and cytokine-treated beta cells, we found that cytokine-treated macrophages had higher basal puromycin incorporation levels, which were significantly reduced by addition of either 2-DG or oligomycin (**Figure 4D**), suggesting increased glycolytic *and* mitochondrial metabolic pathway activity, much like M(IL-4) BMDMs. However, we also observed higher expression of CD86 and lower expression of CD301 in cytokine-treated islet macrophages (**Figures 4E, F**). Cytokine-treated CD86^+^ islet macrophages showed higher basal puromycin incorporation and sensitivity to both glycolysis inhibition by 2-DG and mitochondrial ATP synthesis by oligomycin, determined by lower protein synthesis rates (**Supplementary Figure 3A**). CD86^+^ islet macrophages thus present characteristics of increased glycolytic and mitochondrial metabolic pathway activity, unlike classically activated BMDMs which shut down mitochondrial ATP production. By contrast, CD301^+^ islet macrophages, representing a small fraction of the total islet-resident macrophage population, showed no treatment-dependent changes (**Supplementary Figure 3B**).

### Beta cell death and dysfunction alters metabolic features of pancreatic islet cell populations

**Figure 5:**
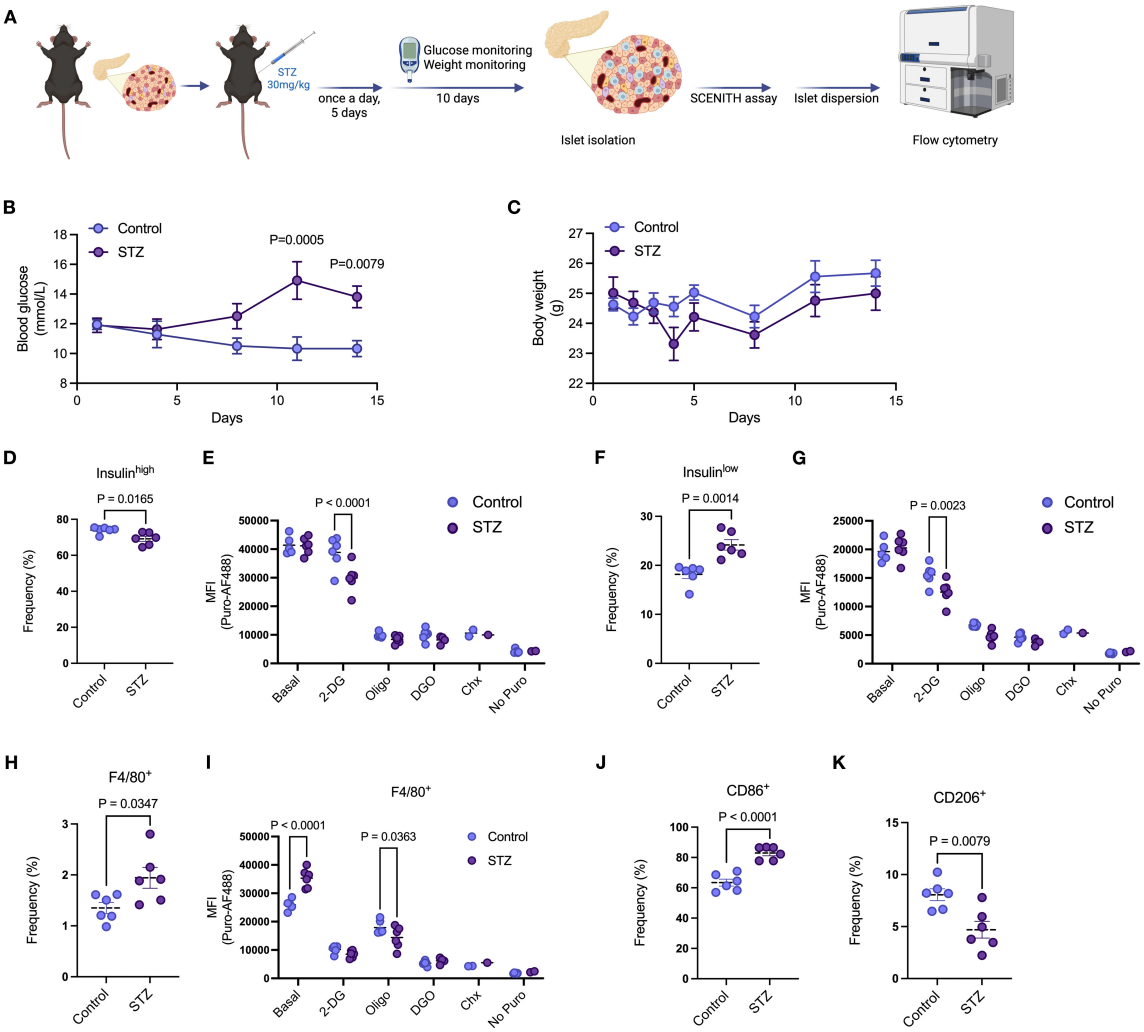
Beta cell death and dysfunction alters metabolic features of pancreatic islet cell populations. **(A)** Schematic representation of multiple low-dose STZ-induced mild beta cell death. C57/BL6 mice were treated with 30mg/kg of STZ for 5 consecutive days with daily body weight monitoring. Blood glucose was monitored every 3 days with no fasting. After the last injection, body weight was also monitored every 3 days. Islets were then isolated from mice, and the puromycin-based assay was performed. **(B)** Blood glucose levels of mice treated with vehicle or STZ. **(C)** Body weight of mice treated with vehicle or STZ. **(D)** Frequency of insulin^high^ beta cells in isolated islets of mice treated with vehicle or STZ. **(E)** MFI of puromycin in insulin^high^ beta cells in isolated islets of mice treated with vehicle or STZ at basal levels, following 2-DG, oligomycin (oligo), 2-DG + oligomycin (DGO), and cycloheximide (Chx) treatments. **(F)** Frequency of insulin^low^ beta cells in isolated islets of mice treated with vehicle or STZ. **(G)** MFI of puromycin in insulin^low^ beta cells in isolated islets of mice treated with vehicle or STZ at basal levels, following 2-DG, oligomycin (oligo), 2-DG + oligomycin (DGO), and cycloheximide (Chx) treatments. **(H)** Frequency of islet macrophages (F4/80^+^) from isolated islets of mice treated with vehicle or STZ. **(I)** MFI of puromycin in islet macrophages (F4/80^+^) from isolated islets of mice treated with vehicle or STZ at basal levels, following 2-DG, oligomycin (oligo), 2-DG + oligomycin (DGO), and cycloheximide (Chx) treatments. **(J, K)** Frequency of CD86^+^ and CD206^+^ islet macrophages (F4/80^+^) from isolated islets of mice treated with vehicle or STZ. Mouse islets n= 6 biological replicates per group. Results are the mean +/- SEM. P-values were calculated by Two-Way ANOVA **(B, C, E, G, I)** with Sidak’s multiple comparison test and t test **(D, F, H, J, K)**.

We then induced beta cell death and dysfunction by administering multiple low-dose injections of STZ (daily for 5 consecutive days) and harvested the islets 10 days after the last injection (**Figure 5A**). Over the duration of the experiment (day zero to 15), mice in the STZ group developed mild hyperglycemia, with no change in body weight between mice in control and STZ groups (**Figures 5B, C**). We then performed the puromycin-based assay in the presence of metabolic inhibitors prior to islet dispersion. As described by others (28), we detected two distinct beta cell populations, characterized as Insulin^high^ or Insulin^low^ (**Supplementary Figure 4A**). STZ led to a reduction in the proportion of Insulin^high^ beta cells and an accumulation of Insulin^low^ beta cells (**Figures 5D, F**). We found that Insulin^high^ beta cells from our control group showed no response to 2-DG treatment, whereas STZ-treated Insulin^high^ beta cells had significantly lower protein synthesis rates (**Figure 5E**). We observed lower puromycin MFI in both control and STZ-treated Insulin^low^ beta cells in relation to basal puromycin uptake (**Figure 5G**). The STZ-treated group was even more sensitive to 2-DG, having significantly lower protein synthesis rates compared to control (**Figure 5G**). These data suggest higher reliance on glycolysis by Insulin^low^ beta cells, which is exacerbated following STZ treatment.

Multiple low-dose STZ also led to increased frequency of both CD3^+^ T cells (**Supplementary Figures 4C, D**) and islet macrophages (**Supplementary Figure 4B**, **Figure 5H**). While there were no significant differences in response to metabolic inhibitors in CD3+ T cells between control and STZ treated mouse islets (**Supplementary Figure 4E**), in islet macrophages STZ led to higher puromycin incorporation at basal levels and increased response to oligomycin, indicating lower protein synthesis rates compared to control (**Figure 5I**). Similar to our *in vitro* stress model using pro-inflammatory cytokines, STZ-induced beta cell death promoted higher expression of CD86 and lower expression of CD206 in islet macrophages (**Figures 5J, K**). Both CD86^+^ and CD206^+^ macrophages exhibited higher basal puromycin incorporation rates following STZ treatment but only CD86^+^ macrophages in the STZ group revealed exacerbated sensitivity to oligomycin compared to control (**Supplementary Figures 4F, G**). These data highlight differential metabolic pathway utilization by specific islet macrophage subtypes after STZ-induced beta cell toxicity.

## Discussion

Immune cell function is influenced by and even dependent on metabolic reprogramming. Tissue-resident immune cell metabolism is still poorly understood, in part due to access difficulty and low frequency of tissue resident-immune cells. There is an urgent need to adapt and improve techniques that can accurately assess cellular metabolic demands at a single-cell level. Here we demonstrate adaptation of the puromycin-based SCENITH protocol to analyze pancreatic islet immune cell metabolism at the single cell level.

Importantly, even though SCENITH is designed for rare cell populations coming from heterogenous environments, it is still challenging to recover sufficient islet-resident immune cells for complete analysis by SCENITH, including basal measurements plus treatments with 2-DG, oligo, DGO, cycloheximide, and no puromycin control, while maintaining biological replicates without pooling samples. We therefore applied SCENITH principles to develop an approach for characterization of islet-resident immune cell profile of energy production. When the experimental sample is so limited that calculations of glucose and mitochondrial dependence, as well as glycolytic and fatty acid/amino acid oxidation capacity are not viable (20), simply determining the rate of puromycin incorporation with either antibodies or newly-developed click-chemistry compatible puromycin analogues (17) (as a surrogate measure of protein synthesis) following disruptions of glycolysis (2-DG) or ATP synthase (oligo) can provide insight into the reliance on glycolysis or mitochondrial respiration for cellular processes in disease models.

Our assay has potential for overcoming barriers for study of pancreatic islet immune cells, major players in the pathogenesis of both type 1 and type 2 diabetes (33, 34). Tissue-resident immune cells are naturally rare in abundance despite playing essential roles in tissue surveillance and homeostasis (17, 35) and playing key roles in beta cell function, dysfunction, and death in response to systemic and local metabolic and inflammatory stressors such as autoantibodies, hyperglycemia, and islet amyloid (33, 36). Local immune cell activation is rooted in environmental signals and adaptations to niche environments that dictate immune cell phenotype (37, 38). For instance, in mice administered high fat diet, adipose tissue-resident macrophages upregulate mTOR activity pathway, whereas peritoneal macrophages from the same mice show reduced phosphorylation of mTOR targets (39). Regulatory T cells (Tregs) present tissue-specific transcriptional profiles including, for example, upregulation of genes implicated in lipid processing (40). Invariant natural killer cells (iNKT) in the spleen and in the liver rely on aerobic glycolysis and produce high levels of IFNγ, while adipose tissue iNKT cells have lower glycolytic rates, and are transcriptionally adapted for fatty acid oxidation (41). Islet macrophages respond to stimuli coming from the blood due to their proximity to blood vessels in the islet, and in NOD mice islet macrophages upregulate genes involved in inflammatory processes, cellular response to oxidative stress, and oxidative phosphorylation (32). This assay has potential to further our insight into these processes in health and disease. In addition, recent advances have led to clinical trials of stem cell-derived islets for cell replacement therapy in type 1 diabetes (42, 43). There are still challenges when it comes to recapitulating human beta cell metabolism *in vitro* and *in vivo* using stem cell-derived beta cells, and pseudo-islet optimization by clustering different endocrine cells (44–46) and immune cells may underlie creation of a more mature and functional stem cell-derived islet source. Applying our puromycin-based assay to stem cell derived islets may improve assessment of cell sources prior to transplant in type 1 diabetes.

Our study describes a comprehensive method for probing the metabolic profile of islet-resident immune cells, building on the SCENITH assay while complementing transcriptomic and proteomic approaches. Our approach has the potential for broad use towards enhancing understanding and providing new insights into the changes that occur in islet-resident immune cell metabolic pathways prior to diabetes onset, during disease progression, in response to therapy, and in cell therapy.

### Limitations

The present study utilized isolated islets from individual mice as biological replicates. Due to low islet yield post isolation, in some experiments we prioritized the comparison of basal puromycin incorporation rates with puromycin incorporation following 2-DG or oligomycin, thus, foregoing the condition of 2-DG + oligomycin or cycloheximide control for inhibition of protein synthesis. One way to address this would be to pool islets of age/sex-matched mice of the same background to overcome the challenge of low islet counts. Additionally, this study used human islets derived from a single donor. We acknowledge that an *n* = 1 cannot be considered for statistical relevance. Still, the application of this method using primary human islets from one donor indicates the potential of this method for use in human islet research.

## Data Availability

No omics datasets were generated or made available, but requests to access the original data or reagents in this publication should be directed to ramon.kleingeltink@bcchr.ca.

## Supplementary material

Supplementary Figure 1 Flow cytometry gating strategy for identification of islet cell populations. Insulin^+^ (AF647) beta cells and glucagon^+^ (BV421) alpha cells were gated from live cells (FVD-eF780). Macrophages (F4/80^+^-Sb702) were gated from CD45^+^ (BV605) immune cells in live cells (FVD-eF780). This was done after exclusion of debris (FSC-A/SSC-A) and doublets (FSC-A/FSC-H).

Supplementary Figure 2 Puromycin-based assay for metabolic profiling of beta cells and macrophages. **(A)** MFI of puromycin in MIN6 cells at basal level and following 2-DG and oligomycin (Oligo) treatments. **(B)** Gating strategy for identification of live BMDMs. Macrophages (F4/80^+^-Sb702) were gated from live cells (FVD-eF780) after exclusion of debris (FSC-A/SSC-A) and doublets (FSC-A/FSC-H). **(C)** MFI of puromycin in BMDMs. BMDMs were left unpolarized (M0) or submitted to 24h polarization in IFNγ+LPS or IL-4. BMDMs n=3 biological replicates. Results are the mean +/- SEM. P-values were calculated by One-Way ANOVA **(B)**.

Supplementary Figure 3 Distinct protein synthesis rates in cytokine-treated islet macrophages. **(A, B)** MFI of puromycin islet-resident CD86^+^ **(A)** and CD301^+^ **(B)** macrophages (F4/80^+^) from dispersed mouse islets treated *ex vivo* with either DMSO vehicle or cytokines. PS rates (puromycin incorporation) shown at basal levels, following 2-DG, oligomycin (oligo), and cycloheximide (Chx) treatments. Mouse islets n= 3 biological replicates. Results are the mean +/- SEM. P-values were calculated by Two-Way ANOVA **(C, D)** with Sidak’s multiple comparison test.

Supplementary Figure 4 Protein synthesis rates in islet immune cells after beat cell death induced by STZ. **(A)** Representative plot for frequency of insulin^high^ and insulin^low^ beta cells from isolated mouse islets. **(B)** Representative plot for frequency of islet macrophages (F4/80^+^). **(C)** Representative plot for frequency of islet T cells (CD3^+^) and unstained control. **(D)** Frequency of T cells (CD3^+^) isolated islets of mice treated with vehicle or STZ. E) MFI of puromycin in islet T cells (CD3^+^) from isolated islets of mice treated with vehicle or STZ at basal levels, following 2-DG, oligomycin (oligo), 2-DG + oligomycin (DGO), and cycloheximide (Chx) treatments. **(F, G)** MFI of puromycin in islet CD86^+^ **(D)** and CD301^+^ **(E)** macrophages (F4/80^+^) from isolated islets of mice treated with vehicle or STZ at basal levels, following 2-DG, oligomycin (oligo), 2-DG + oligomycin (DGO), and cycloheximide (Chx) treatments. Mouse islets n= 6 biological replicates per group. Results are the mean +/- SEM. P-values were calculated by Two-Way ANOVA **(E-G)** with Sidak’s multiple comparison test and t test **(D)**.
