# Supplementary figures and images for: Assessment of protein synthesis rate enables metabolic profiling of resident-immune cells of the islets of Langerhans
Source: Front Immunol. 2025 Sep 23;16:1662986. doi: 10.3389/fimmu.2025.1662986 (PMC12500629; doi:10.3389/fimmu.2025.1662986)

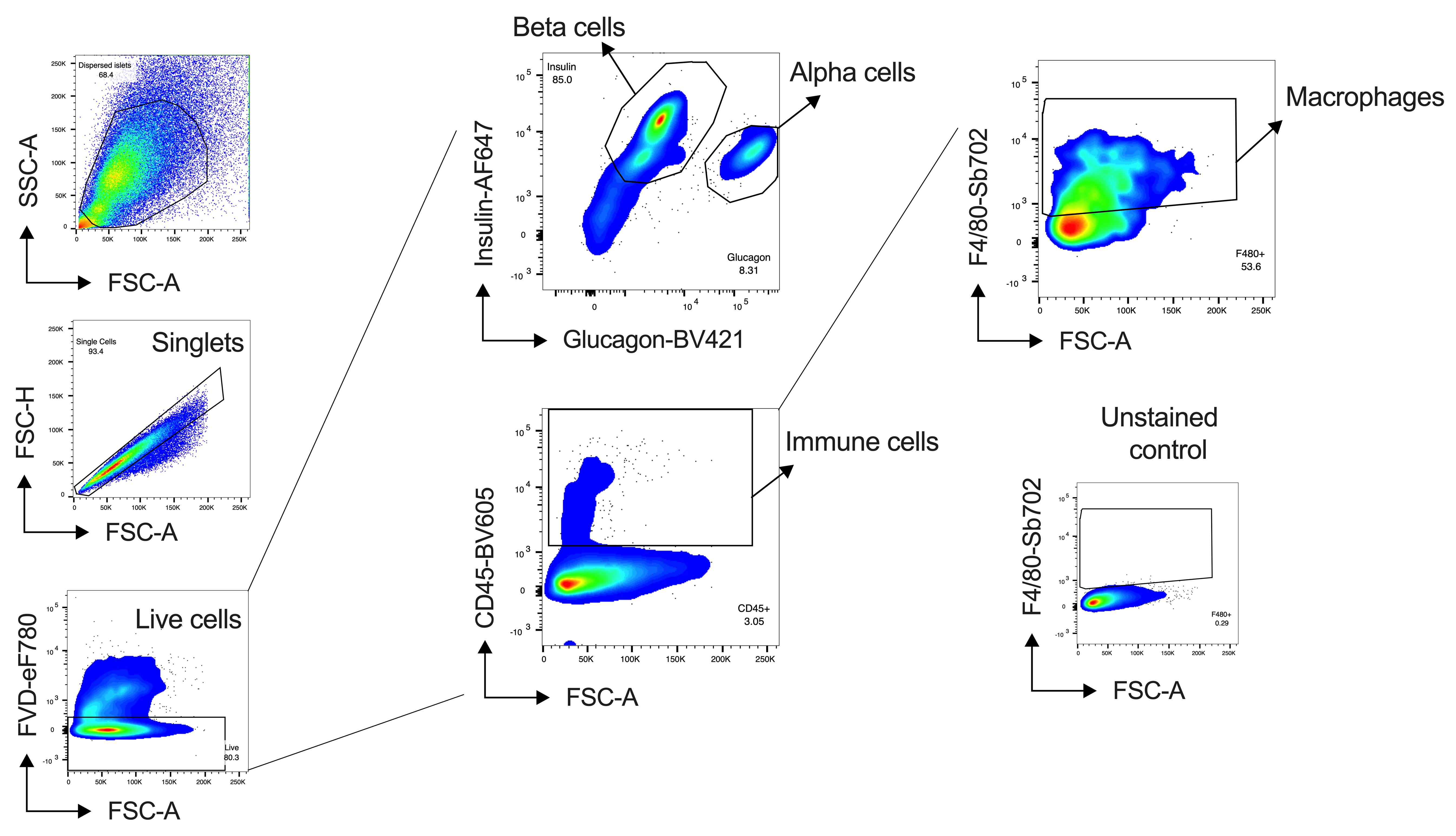

Supplement: Supplementary Figure 1 — Flow cytometry gating strategy for identification of islet cell populations. Insulin+ (AF647) beta cells and glucagon+ (BV421) alpha cells were gated from live cells (FVD-eF780). Macrophages (F4/80+-Sb702) were gated from CD45+ (BV605) immune cells in live cells (FVD-eF780). This was done after exclusion of debris (FSC-A/SSC-A) and doublets (FSC-A/FSC-H). [file Image1.tiff]

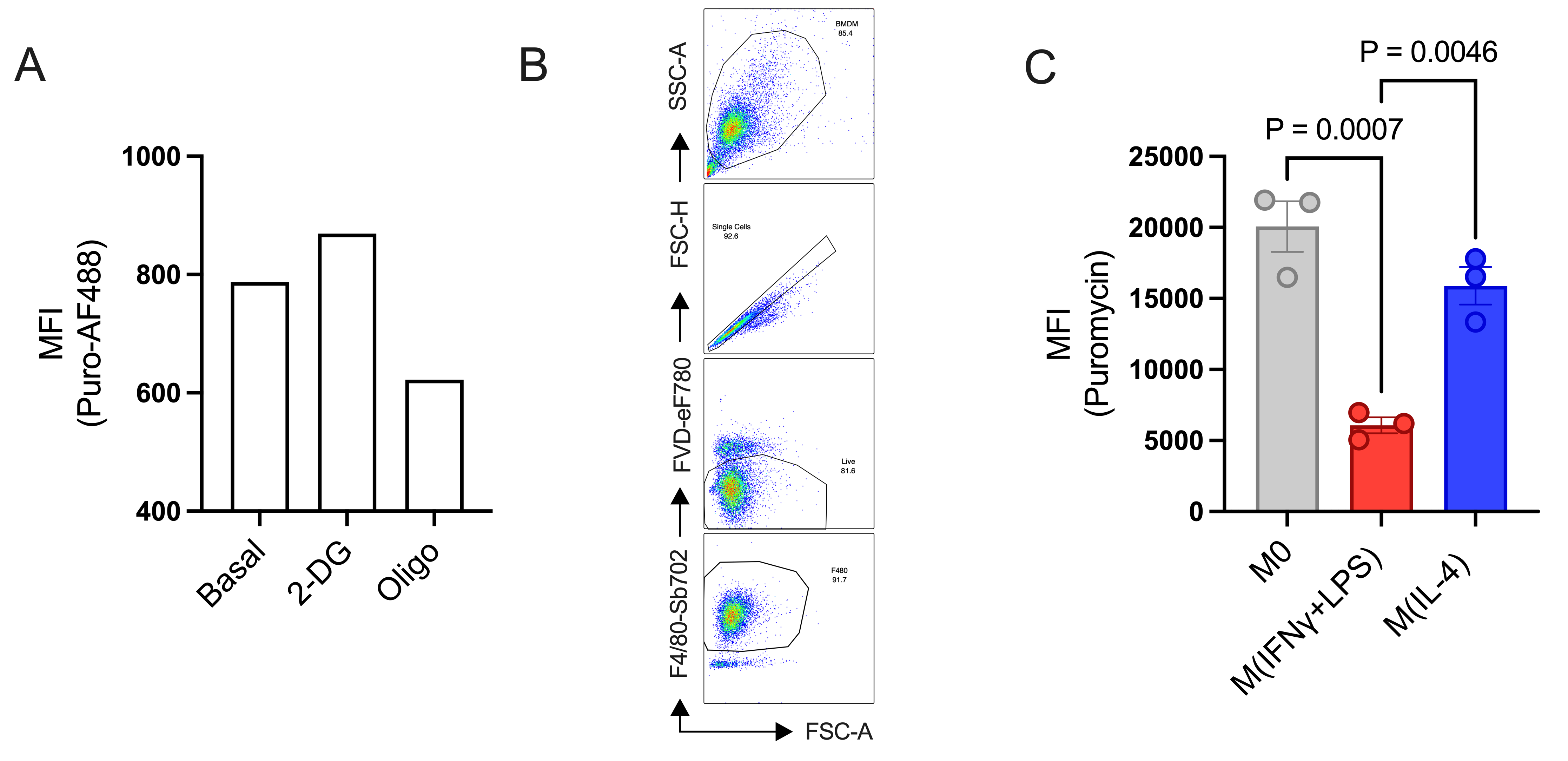

Supplement: Supplementary Figure 2 — Puromycin-based assay for metabolic profiling of beta cells and macrophages. (A) MFI of puromycin in MIN6 cells at basal level and following 2-DG and oligomycin (Oligo) treatments. (B) Gating strategy for identification of live BMDMs. Macrophages (F4/80+-Sb702) were gated from live cells (FVD-eF780) after exclusion of debris (FSC-A/SSC-A) and doublets (FSC-A/FSC-H). (C) MFI of puromycin in BMDMs. BMDMs were left unpolarized (M0) or submitted to 24h polarization in IFNγ+LPS or IL-4. BMDMs n=3 biological replicates. Results are the mean +/- SEM. P-values were calculated by One-Way ANOVA (B). [file Image2.tiff]

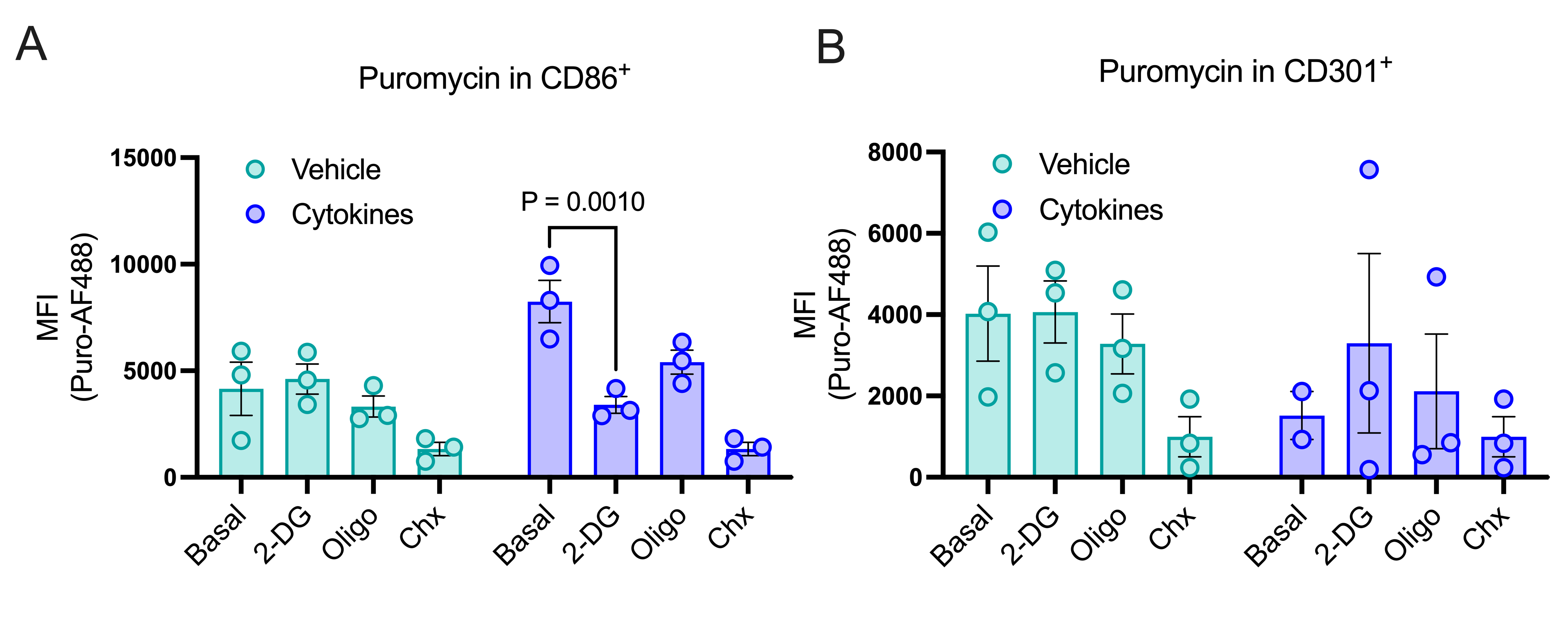

Supplement: Supplementary Figure 3 — Distinct protein synthesis rates in cytokine-treated islet macrophages. (A, B) MFI of puromycin islet-resident CD86+ (A) and CD301+ (B) macrophages (F4/80+) from dispersed mouse islets treated ex vivo with either DMSO vehicle or cytokines. PS rates (puromycin incorporation) shown at basal levels, following 2-DG, oligomycin (oligo), and cycloheximide (Chx) treatments. Mouse islets n= 3 biological replicates. Results are the mean +/- SEM. P-values were calculated by Two-Way ANOVA (C, D) with Sidak’s multiple comparison test. [file Image3.tiff]

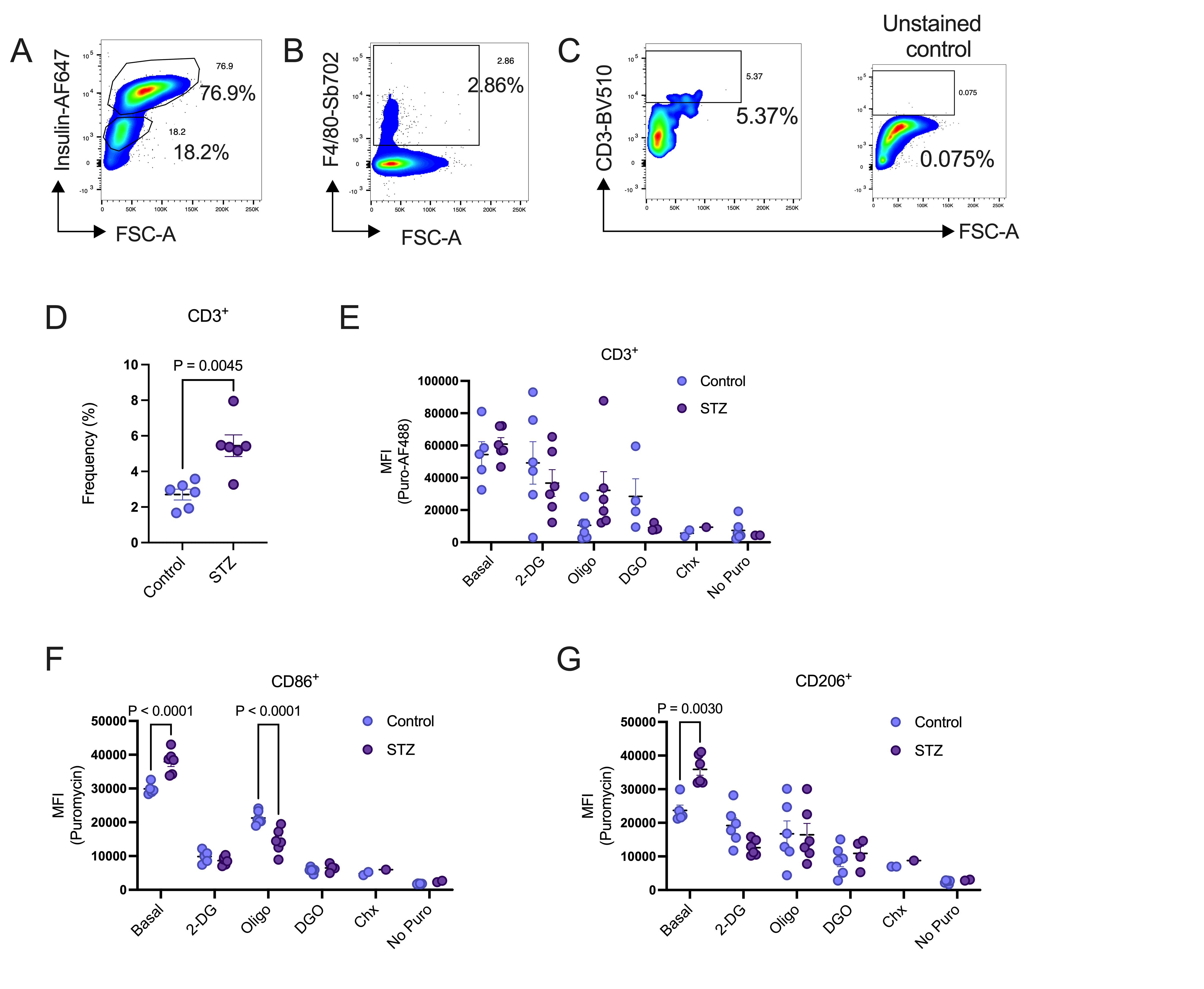

Supplement: Supplementary Figure 4 — Protein synthesis rates in islet immune cells after beat cell death induced by STZ. (A) Representative plot for frequency of insulinhigh and insulinlow beta cells from isolated mouse islets. (B) Representative plot for frequency of islet macrophages (F4/80+). (C) Representative plot for frequency of islet T cells (CD3+) and unstained control. (D) Frequency of T cells (CD3+) isolated islets of mice treated with vehicle or STZ. E) MFI of puromycin in islet T cells (CD3+) from isolated islets of mice treated with vehicle or STZ at basal levels, following 2-DG, oligomycin (oligo), 2-DG + oligomycin (DGO), and cycloheximide (Chx) treatments. (F, G) MFI of puromycin in islet CD86+ (D) and CD301+ (E) macrophages (F4/80+) from isolated islets of mice treated with vehicle or STZ at basal levels, following 2-DG, oligomycin (oligo), 2-DG + oligomycin (DGO), and cycloheximide (Chx) treatments. Mouse islets n= 6 biological replicates per group. Results are the mean +/- SEM. P-values were calculated by Two-Way ANOVA (E-G) with Sidak’s multiple comparison test and t test (D). [file Image4.tiff]
